# Possibilities of a Direct Synthesis of Aluminum Alloys with Elements from Deep-Sea Nodules

**DOI:** 10.3390/ma15134467

**Published:** 2022-06-24

**Authors:** Klára Borkovcová, Pavel Novák

**Affiliations:** Department of Metals and Corrosion Engineering, University of Chemistry and Technology, Prague, Technická 5, 166 28 Prague, Czech Republic; panovak@vscht.cz

**Keywords:** deep-sea nodules, aluminothermy, manganese-aluminum alloy

## Abstract

This work investigated the possibility of the direct preparation of aluminum alloys by aluminothermic reduction of deep-sea nodules with a high excess of aluminum. The process was found to be unable to obtain aluminum alloy, but an aluminum-rich manganese-based alloy was obtained instead, being composed of intermetallics. The alloy was characterized in the as-reduced state, as well as after crushing and sintering in the temperature range of 800–950 °C. The sample sintered at 900 °C was also heat-treated by annealing at 800 °C for 3 h and rapidly cooled. It was observed that with the increasing sintering temperature, the original matrix phase Al_11_Mn_14_ was transformed into a duplex matrix with a structure corresponding to Al_11_Mn_14_ and Al_4_Cu_9_, and this mixture was further transformed to the matrix with the structure corresponding to Al_4_Cu_9_. Furthermore, the mechanical properties and wear resistance of the samples were described. The highest microhardness was reached in the sample, which was annealed after sintering. Sintered samples reached a lower wear rate because of the fragmentation of brittle intermetallics during crushing.

## 1. Introduction

Deep-sea nodules are ores containing manganese oxides, which are enriched mainly with iron, cobalt, nickel and copper. Other minor elements in nodules are zinc, silicon, titanium or magnesium and alkaline earth metals. They occur most often in all oceans at depths of 3000–6000 m with a size of 1–15 cm, but mostly in areas where the rate of sediment accumulation is slow [1,2,3,4]. At the bottom of the sea, nodules occur in continuous fields, in which a certain size of nodules dominates. Deep-sea nodules are formed by concentric layers of iron hydroxides and manganese hydroxides around the core. This nucleus can be a fossilized shark tooth or bone [5,6].

Chemical-composition studies have been conducted since the 1960s, where the National Oceanic and Atmospheric Administration (NOAA) found that the exact chemical composition is determined primarily by water depth, geographic findings and ocean circulation. From these results, the places with the highest incidence of deep-sea nodules were determined, which are mainly the Clarion-Clipperton Fracture Zones (CCZ) in the Pacific Ocean and the Indian Ocean [3,7,8]. According to a Japanese study, the chemical composition of deep-sea nodules was determined arithmetically, where it is stated that manganese is contained in nodules in 17–26%, nickel in 0–54%, copper in 0–32% and cobalt in 0–28% [9]. In the Clarion-Clipperton area, the nodule composition is reported as 25.4% Mn, 6.66% Fe, 1.27% Ni and 1.02% Co. Iron occurs in deep-sea nodules in the form of oxides, hydroxide-oxides and as a hydrated oxide. There are α-FeOOH and γ-FeOOH phases in the nodules, as well as a smaller amount of the magnetite phase. Cobalt is present in nodules between the iron and manganese phases. The content of nickel and cobalt in nodules increases together with the content of manganese. Furthermore, the content of cobalt, titanium and zinc in nodules increases with the content of iron and manganese [9,10,11,12]. There is usually no iron, cobalt and lead in the core of nodules. In the upper shell of nodules, compared to the inner shell of nodules, there is a larger amount of iron, cobalt and lead and a smaller amount of copper and nickel [8].

Deep-sea nodules are very porous solids. This leads to a high moisture content in the nodules. This has a negative effect on the cost of their further processing, when the nodules have to be dried for a very long time. Another important factor besides porosity is their fragility. However, due to their fragility, less energy is needed for crushing and grinding [13].

Processes for extraction of metals from deep-sea nodules can be divided into two categories, namely pyrometallurgical processes and hydrometallurgical processes. Deep-sea nodules contain up to 40 wt. % humidity. By transporting them to ports, it is possible to reduce the humidity of nodules to 15 wt. %. However, even such increased humidity is undesirable for pyrometallurgy; therefore, it is not used for obtaining metals of interest from nodules by purely pyrometallurgical processes due to the high amount of thermal energy that is necessary to remove the water from nodules. The pyrometallurgical process can be used only for the nodules that contain at least 40 wt. % of manganese. Hydrometallurgical processes involve leaching in acids and bases with reduction with or without it. Another alternative is leaching at high temperatures without the use of a reducing agent. In these processes, manganese and iron dissolve together with copper, nickel and cobalt. Combined pyro-hydrometallurgical processes for the recovery of metals from deep-sea nodules consist of pyrometallurgical and hydrometallurgical processes. Pyrometallurgical processes precede hydrometallurgical processes and their goal is to convert the metals of interest into a soluble form. Such technologies include processes at higher temperatures, which may be chlorination, sulfation or melting. These processes are followed by hydrometallurgical ones, which use leaching and subsequent electrolysis to obtain pure metal [13,14,15].

However, leaching processes consume a large amount of leaching agents, and the resulting solution has a very diverse chemical composition. The individual elements are thus further removed by specific reactions, such as copper extraction using active powdered sulfuric anhydride; nickel and cobalt extraction using active sulfur together with manganese or MnO_2_ extraction from ammonia; and where filtration is used, washing the filter with manganese hydroxide, drying in rotary kilns and briquetting. A more sophisticated way is the selective extraction of metals of interest in acids or bases [16,17]. This selective extraction of metals of interest in acids is achieved by the use of various types of reducing agents, where in the latest study, steel scrap is repeatedly used as a reducing agent for manganese recovery [18,19].

As the methods for obtaining metals from nodules are very complex, a new approach has been proposed. It is based on the nonseparation of individual elements from nodules and the use of nodules as a whole, when a completely new alloy is formed. Since nodules contain a high percentage of MnO_2_ and the process of aluminothermic reduction to reduce MnO_2_ is a commonly used process, a process for treating deep-sea nodules by aluminothermic reduction has been proposed [16], which was used also in this work. In metallothermic reduction, metal is used as a reducing agent. Such reducing agents are metals that have a higher affinity for oxygen. Metallothermic reduction efficiency is not 100%. The metallothermic reaction is strongly exothermic and rapid. The heat released in this reaction depends on the reducing agent selected. It is generally stated that the stronger the reducing agent, the more heat is released during the matrix reduction. Aluminum is the most common when used for metallothermic reduction. Aluminothermic processes are used in the production of manganese, chromium and also for the production of carbon-free ferroalloys. The advantages of aluminothermy mainly include the speed of the reaction, the absence of the need for a heat source, easy adaptation of the reaction to different productions, low investment costs and no CO_2_ emissions. The disadvantage of aluminothermy is the higher price of aluminum [20,21,22,23,24].

Processes for recovering metals from deep-sea nodules are very complex, so a new process for recovering metals from nodules—aluminothermy—has been proposed. It is commonly used to reduce MnO_2_, which is abundant in nodules. This work aims to test the applicability of aluminothermy for direct production of aluminum alloys, containing the elements from deep-sea nodules. The work deals with the mechanical properties, microstructure and chemical composition of the sintered and annealed alloys, and because of their microhardness the tribological properties were also tested.

## 2. Materials and Methods

The deep-sea nodules were crushed to powder with a particle size under 125 µm. The chemical composition of the nodules was measured by X-ray fluorescence spectrometer (XRF, Axios, PANanalytical, Almelo, The Netherlands) and it is presented in the Table 1. One part of the crushed nodules was roasted in an electric resistance furnace (Martinek, Kladno, The Czech Republic) at 250 °C in the air, the other part at 500 °C. The roasting at 250 °C was used for drying of the crushed nodules, while the 500 °C was applied to change the oxidation state of manganese from MnO_2_ to Mn_3_O_4_. Both parts were roasted for 2 h. A mixture in a weight ratio of 1:1 was prepared from the roasted nodules.

Using equations based on the Ellingham diagram, the stoichiometric amount of aluminum required for aluminothermic reduction was calculated [22]. In the previous work [22] there were aluminothermically prepared samples with stoichiometric amounts of aluminum, 10% excess of the aluminum over stoichiometry and 20% excess of aluminum over stoichiometry. In this work, the samples were prepared aluminothermically with 100% excess of aluminum over stoichiometry. The main idea for the high excess of aluminum was to prepare the aluminum-based alloy directly, because the recent work [22] revealed that the aluminum content in the alloy increases with the Al amount in the reaction mixture. The batch of nodules was 500 g, while the amount of aluminum used for the reduction was 229 g. The aluminothermic reactions were initiated by the ignition mixture composed of aluminum powder, sodium peroxide and magnesium metal flakes. After the reaction was completed, metallic alloy and slag were obtained by mechanical separation.

The phase transformations were measured by differential thermal analysis by the means of TG-DTA (Thermogravimetry Differential Thermal Analysis) Setsys Evolution device (Setaram, Caluire-et-Cuire, France) in an argon atmosphere in an alumina crucible. DTA was carried out in the temperature range of 0–1200 °C with a heating rate of 30 K/min.

After aluminothermic reductions, the metal was crushed and the powder consolidated by spark plasma sintering (SPS) using HP D10 (FCT Systeme, Rauenstein, Germany) device. The sintering was carried out at 900 °C for 10 min, using the pressure of 48 MPa and heating rate of 300 K/min, while the controlled cooling rate was 50 K/min. The diameter of the sintered sample was 20 mm. The material obtained by sintering at 900 °C was also heat-treated by annealing at 800 °C for 3 h, followed by water quenching. One sample was sintered at 950 °C for 10 min, using the same pressure and heating rate as it is given above.

The phase composition of materials was determined by powder X-ray diffraction (XRD). Data in Bragg–Brentano geometry were collected on a X’Pert3 Powder diffractometer using CuKα radiation. After etching the sample with Keller’s reagent (2 mL HF, 3 mL HCl, 5 mL HNO_3_ and 190 mL H_2_O), the microstructure was observed using an Eclipse MA200 metallographic optical microscope (Nikon, Tokyo, Japan) and by scanning electron microscope (SEM) VEGA 3 LMU (TESCAN, Brno, Czech Republic). The scanning electron microscope equipped with energy-dispersive spectrometer (EDS) X-max 20 mm^2^ detector (Oxford Instruments, High Wycombe, UK) was applied.

In addition to the phase analysis and microstructure evaluation, the samples were characterized from the viewpoints of mechanical properties (microhardness), as well as the tribological behavior (wear rate and friction coefficient). Microhardness was measured by the Vickers method at a load of 100 g (HV 0.1), which corresponds to 0.98 N. The microhardness was measured five times on each sample and the average values was calculated. To confirm the hardness trend, the hardness was determined once more at a load of 10 g. The wear resistance was measured on a TriboTester tribometer (Tribotechnic, Clichy, France) using the ball-on-disc tribometer in linear reciprocal mode (*e* excenter of 5 mm). In this case, the “ball” was made of alumina (α-Al_2_O_3_) and it had 6 mm in diameter and the “disc” was a sample. No lubricant was used in the process. The sliding distance (*l*) in this process was 20 m and a normal force (*F*) of 5 N was used. The wear rate (*w* [mm^3^ N^−1^ m^−1^]) was calculated from the equation (1), where we take in mind the wear track section area (*A* [mm^2^]).

(1) $$w=\frac{A\cdot e}{F\cdot l}$$

## 3. Results

### 3.1. Microstructure and Phase Composition of the Alloy in the Reduced State

#### 3.1.1. Chemical Composition of the Aluminothermically Prepared Alloy

Table 2 shows the chemical composition of sample in the reduced state with 100% excess aluminum over stoichiometry measured by X-ray fluorescence (XRF). From the results of XRF analysis, it is evident that the metal part contains the most manganese, which is later confirmed by the result of X-ray diffraction analysis of this sample, where manganese is present in all three phases. Another element of the metal phase of the measured XRF is aluminum. It occurs mainly in the Al_11_Mn_14_ phase, but is also contained in the phase with the structure corresponding to Al_2_MnNi. Furthermore, it is clear from the table that the sample prepared aluminothermically also contains, in addition to manganese and aluminum, a large amount of iron, silicon, nickel and copper.

#### 3.1.2. Alloy with 100% Excess of Aluminum over Stoichiometry in the Reduced State

**Figure 1:**
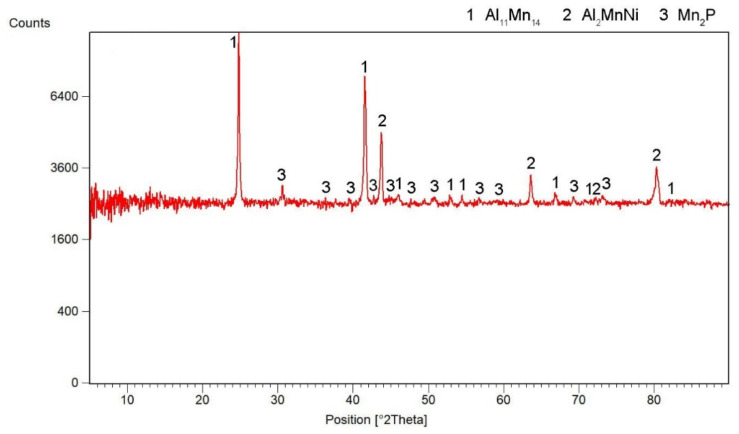
X-ray diffraction pattern of a metal sample in the reduced state.

**Figure 2:**
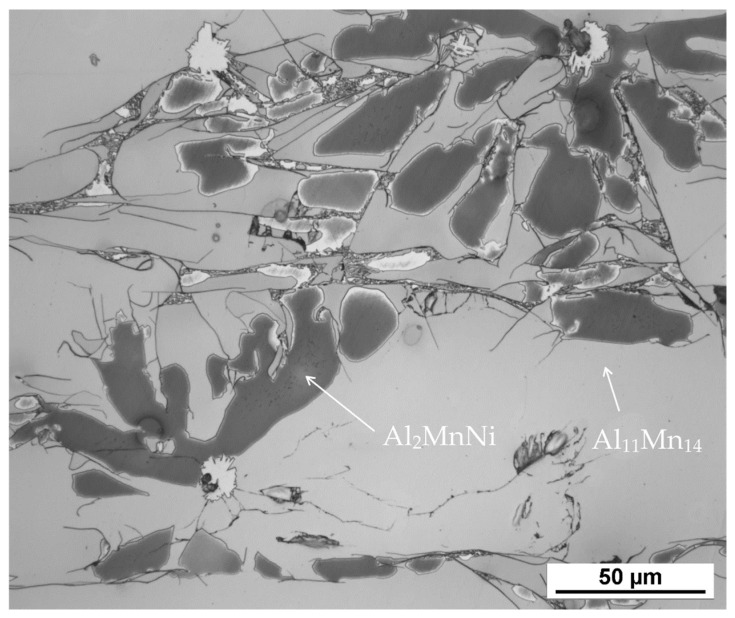
The microstructure of alloy with 100 % excess of aluminum over stoichiometry in the reduced state.

According to X-ray diffraction analysis (Figure 1), the metal sample in reduced state contains three phases, which are Al_11_Mn_14_, Al_2_MnNi and Mn_2_P. The Al_11_Mn_14_ phase (tetragonal, P4/mmm space group, a = 3.940 × 10^−10^ m, c = 3.580 × 10^−10^ m) appears to be very bright in the structure of Figure 2 and is most abundant in the sample [26]. Its chemical composition will be discussed below. The phase having the structure corresponding to Al_2_MnNi phase (cubic, Pm-3 m space group, a = 2.929 × 10^−10^ m) is dark in the optical micrograph and reaches smaller dimensions than the previous phases. In the diffraction pattern in Figure 1, the intensities of the diffraction lines belonging to this phase are lower than the ones of the Al_11_Mn_14_ phase, which corresponds to Figure 2 with a lower representation of this phase. The third phase in the XRD pattern, Mn_2_P (hexagonal, P-63 m space group, a = 6.08 × 10^−10^ m, c = 3.46 × 10^−10^ m) has the lowest intensity of the peaks in Figure 1. However, it is not visible in Figure 2 due to the given magnification. Further in the text, for the SEM microstructure with higher magnification, this phase is visible.

**Figure 3:**
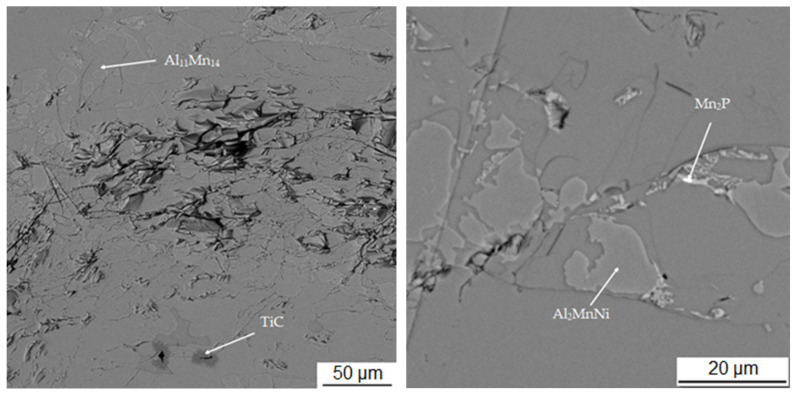
Microstructure of sample with 100% excess of aluminum over stoichiometry in the reduced state.

**Figure 4:**
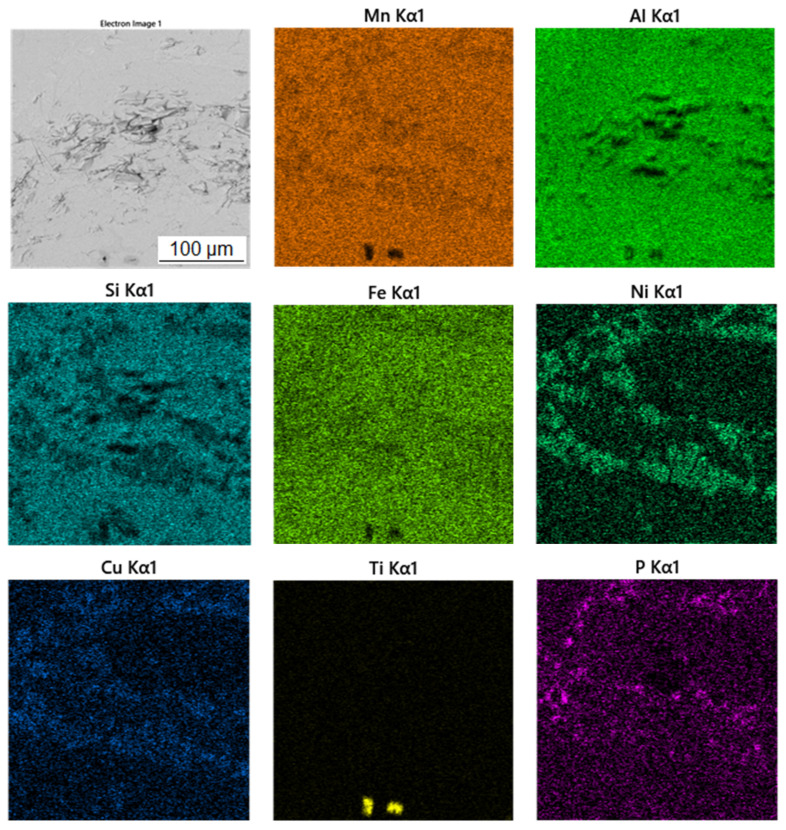
EDS map of the distribution of the elements of the sample in the reduced state.

Table 3 shows the chemical composition of four phases of sample in the reduced state. In Figure 3, showing the SEM micrograph in backscattered electrons (BSE) mode of the sample in the reduced state, the first phase is dark grey and the EDS data show that it contains the most manganese (37.5 at.%) and aluminum (38.4 at.%). The chemical composition of this phase determined by EDS analysis confirms this phase as the Al_11_Mn_14_ matrix phase. It is clear from the element maps in Figure 4 that silicon and manganese are most common in this phase. Silicon generally occurs in this alloy mainly in the Al_11_Mn_14_ phase and it binds its presence to areas with a higher manganese content; it does not bind to the phase that is rich in nickel.

The second phase is light grey in Figure 3 and differs from the first phase in that it contains 5.8 at.% nickel; the amount of manganese is lower compared to the first phase (32.1 at.%). Other elements contained in this phase are aluminum (43.5 at.%) and silicon (7.8 at.%). The results of the EDS analysis show the high amount of nickel in the phase with the structure corresponding to Al_2_MnNi (Figure 4).

The third phase is very bright in detail in Figure 3 and has a high content of manganese (54.9 at.%) and phosphorus (19.8 at.%). Since their higher content then other phases, this phase was identified as Mn_2_P. The occurrence of this phase was confirmed by EDS analysis and X-ray diffraction analysis and was captured by spot analysis only at higher magnification, as it reached the size of approximately 2 μm. The regions with high phosphorus content, i.e., the Mn_2_P particles, can be also observed on the element map in Figure 4.

The last phase recognized by EDS analysis has a high content of titanium (35.7 at.%), carbon (49.6 at.%) and low contents of manganese (3.7 at.%) and aluminum (5.8 at.%). This phase was not identified by X-ray diffraction analysis, but from the EDS point analysis (Table 3) and elemental map (Figure 4) it can be determined that it is titanium carbide based on the high proportion of carbon and titanium. Cracking is visible around this phase in Figure 3. This is probably due to the different thermal expansion of the carbide and the surrounding phases, as a result of which cracks formed during the cooling of the alloy after reduction.

### 3.2. Differential Thermal Analysis of the Reduced Metal Alloy

**Figure 5:**
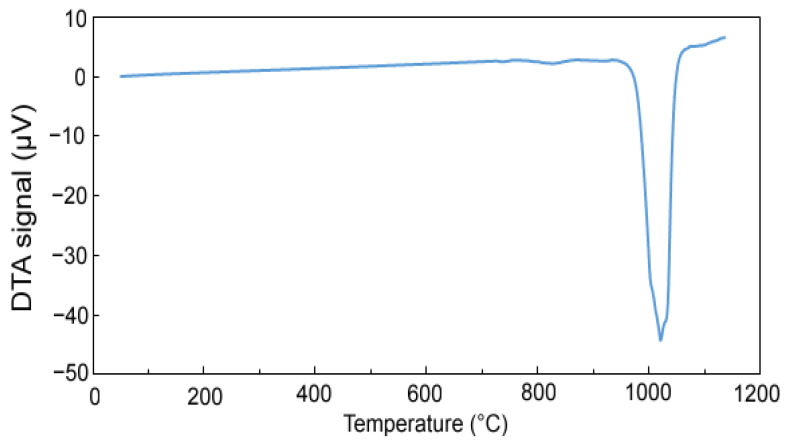
DTA heating curve of sample with 100% excess of aluminum over stoichiometry in the reduced state.

Figure 5 shows the DTA heating curve to determine the phase transformations during heating. At a temperature of 960 °C, an endothermic effect starts and reaches its minimum at a temperature of 1020 °C, then the DTA signal values return to a stable level. This endothermic peak is associated with the formation of a melt, which is most likely associated with a eutectic transformation. Based on these results, maximal sintering temperature was determined to be approx. 950 °C.

### 3.3. Microstructure and Phase Composition of the Alloy after Sintering Process and Heat Treatment

**Figure 6:**
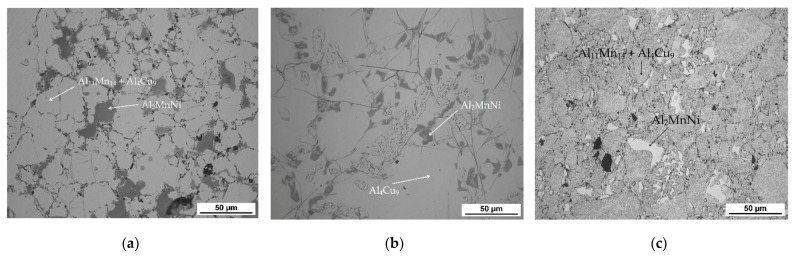
The microstructure of alloy with 100% excess of aluminum over stoichiometry: (**a**) sintered at 900 °C; (**b**) sintered at 950 °C; (**c**) sintered at 900 °C and then heat-treated.

**Figure 7:**
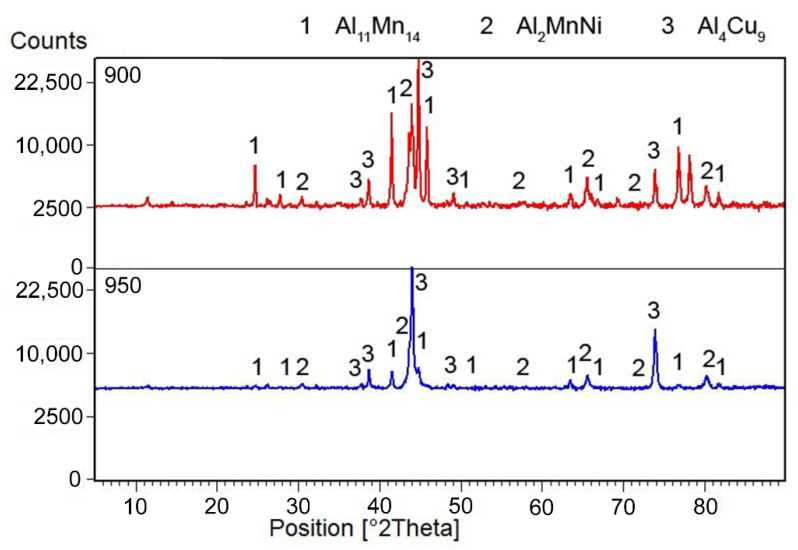
X-ray diffraction pattern of a metal sample sintered at 900 and 950 °C.

The microstructure of the alloys of all three sintered samples is shown in Figure 6. Grinding and subsequent sintering reduce the grain size (Figure 6a–c) because the particles of intermetallics are fragmented during the grinding process. Figure 6b and the XRD pattern in Figure 7 show the dominant phase (Al_11_Mn_14_), the darker phase (Al_2_MnNi) and the formation of new very small particles in the matrix phase. According to XRD, this phase has the structure of Al_4_Cu_9_ (cubic, P-43 m space group, a = 8.704 × 10^−10^ m), even though its chemical composition is probably strongly different, because the content of copper in the alloy is rather low. The alloy sintered at 950 °C (Figure 6b) has lower porosity than the one sintered at 900 °C (Figure 6a), because the partial melting occurred during sintering. Even though the temperature was set according to DTA to be below the observed eutectic temperature, the material was molten during the process, the possible reasons for which are as follows:

- The actual temperature in the sample may be higher than the temperature measured by the optical pyrometer, because the temperature in the SPS is not measured in the sample, but in the punch cavity at a distance of about 2 mm from the sample surface.
- The temperature rose slightly above the set temperature and exceeded this limit locally.

Therefore, even though sintering at 950 °C produces lower porosity, its use cannot be recommended.

#### 3.3.1. Alloy Sintered at 900 °C

**Figure 8:**
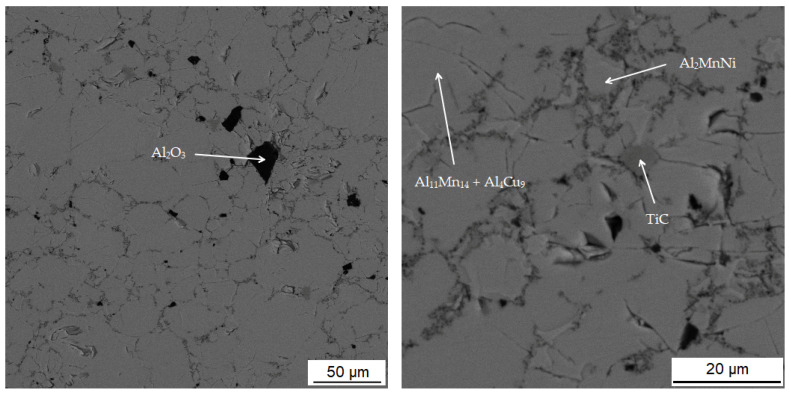
Microstructure of sample with 100% excess of aluminum over stoichiometry sintered at 900 °C.

**Figure 9:**
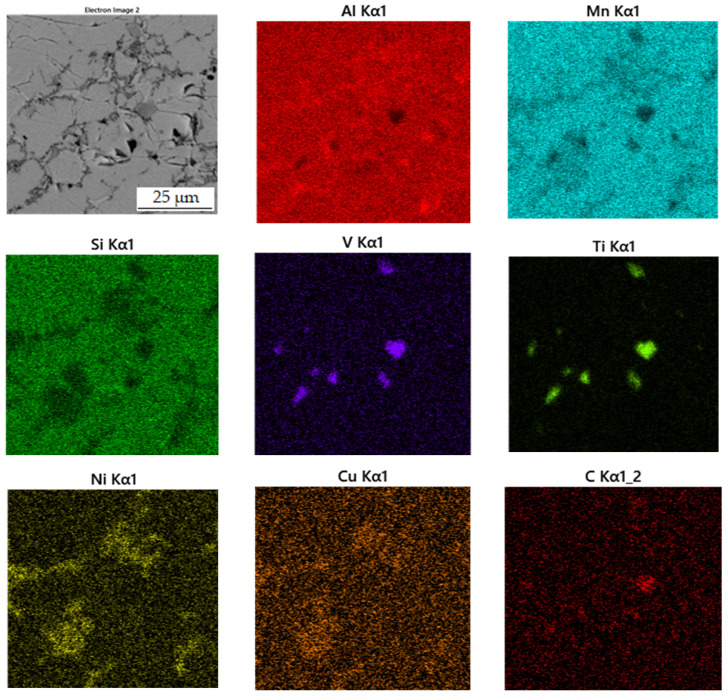
EDS map of the distribution of the elements in sample sintered at 900 °C.

In Table 4 there is a composition of phases of the sample sintered at 900 °C. The first phase contains 37.2 at.% manganese, 39.7 at.% aluminum and 14.0 at.% silicon. In Figure 8, this phase appears grey. From the distribution of the elements in Figure 9, it can be seen that the elements aluminum, manganese and silicon bind to the matrix phase. In addition, a relatively large amount of copper is present in the regions of the matrix phase, which in the previous samples, however, binds mainly to the regions of the phase with the structure corresponding to Al_2_MnNi. It is also evident from the micrograph that the matrix is not homogeneous, but a new phase begins to form in it. Furthermore, the results of X-ray diffraction analysis show that a newly formed phase with a structure corresponding to Al_4_Cu_9_ occurs in a high amount in the sample. Therefore, it is highly probable that this sample contains a two-phase matrix with a structure corresponding to Al_11_Mn_14_ and Al_4_Cu_9_.

The second phase appears lighter in the microstructure image than the matrix phase. It also reaches smaller dimensions than the matrix phase and is surrounded by a matrix. This phase contains 31.2 at.% manganese, 44.0 at.% aluminum and 5.7 at.% nickel. Due to the higher amount of nickel in this phase determined from the EDS analysis and the results of the X-ray diffraction analysis, it follows that it is the Al_2_MnNi phase. From the element maps in Figure 9 it is clear that nickel, and to a lesser extent also copper, are bound to this phase.

The third phase is dark gray in the microstructure image and contains the most carbon (44.4 at.%) followed by titanium (24.5 at.%), the same amount of manganese, aluminum (10.9 at.%) and vanadium (3.3 at.%). From the high content of carbon, titanium and vanadium, which is also in Figure 9 of the element maps, it can be concluded that this is the MC carbide phase, based mostly on titanium. The picture of the microstructure shows that this phase reaches a size of up to 10 µm. Cracks form around this phase, which is most probably due to the different thermal expansion of the carbide and the surrounding phases.

The last phase in this sample appears to be the darkest one in Figure 8 acquired in backscattered electron (BSE) mode and contains the highest amount of oxygen (42.0 at.%), followed by high amount of aluminum (35.5 at.%). From these values, it is determined that it is the Al_2_O_3_ phase. This phase was probably incorporated into the sample as a slag residue.

#### 3.3.2. Alloy Sintered at 950 °C

**Figure 10:**
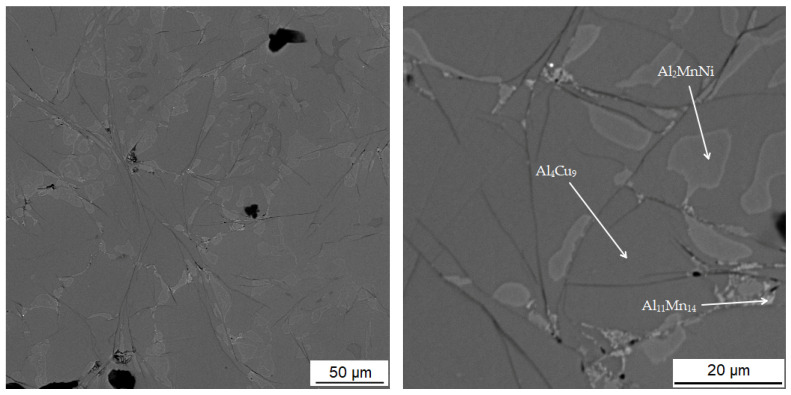
Microstructure of alloy with 100% excess of aluminum over stoichiometry sintered at 950 °C.

**Figure 11:**
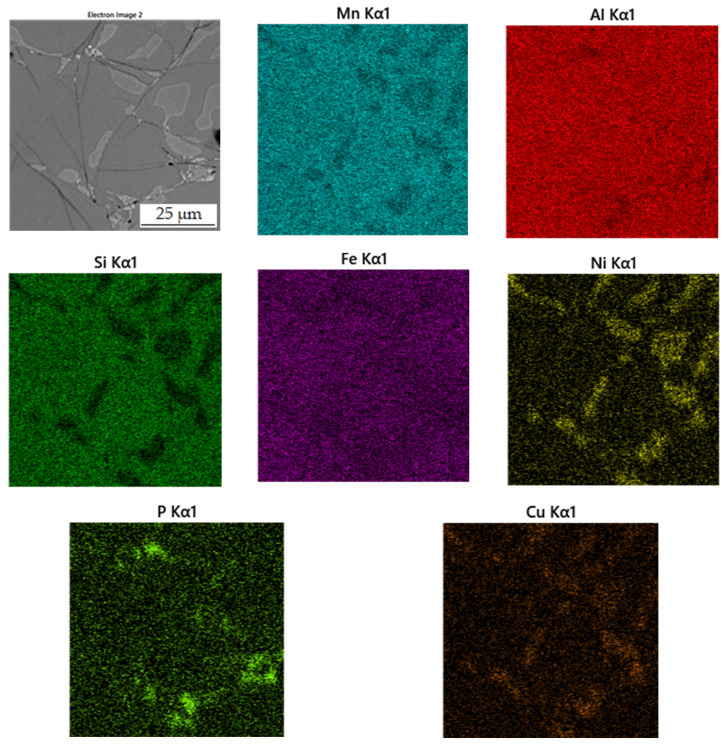
EDS map of the distribution of the elements in alloy sintered at 950 °C.

Table 5 summarizes the chemical composition of three phases for material sintered at 950 °C. The sample thus sintered has a matrix phase, which is dark grey in Figure 10, with a composition of 39.0 at.% manganese, 37.5 at.% aluminum and 14.8 at.% silicon. X-ray diffraction analysis shows that as the sintering-process temperature increases, a new matrix phase with a structure corresponding to Al_4_Cu_9_ is formed, which replaces the original matrix phase. This phase arises at the expense of the other two phases contained in the sample. This phase has a structure corresponding to said Al_4_Cu_9_ phase, but a completely different chemical composition, as evidenced by the table of chemical composition of individual phases (Table 4) and Figure 11 with a map of the distribution of elements where very little copper is contained.

The second phase, light grey in Figure 10, contains 42.7 at.% aluminum, 31.3 at.% manganese, 7.4 at.% silicon and 6.4 at.% nickel. Due to higher content of nickel in this phase, it was identified as the Al_2_MnNi phase based on the results of EDS analysis and X-ray diffraction analysis. It is clear from the distribution of elements that both nickel and copper bind to this phase. The iron is relatively evenly distributed between the two phases present.

The last phase detected by EDS analysis is the phase that contains 39.4 at.% manganese, 37.0 at.% aluminum and 14.7 at.% silicon. According to X-ray diffraction analysis and EDS analysis, it was determined to be the Al_11_Mn_14_ phase.

Alloys sintered at 900 °C and 950 °C were prepared in a very similar way, with the only difference being temperature of sintering, and yet these alloys are very different, mainly in phase composition. The main difference is in the phase composition, where there is a matrix with large predominance of the phase identified as Al_4_Cu_9_ in the sample sintered at 950 °C, while there is a majority of Al_11_Mn_4_ after sintering at 900 °C.

#### 3.3.3. Alloy Sintered at 900 °C and Heat-Treated by Annealing at 800 °C for 3 h

**Figure 12:**
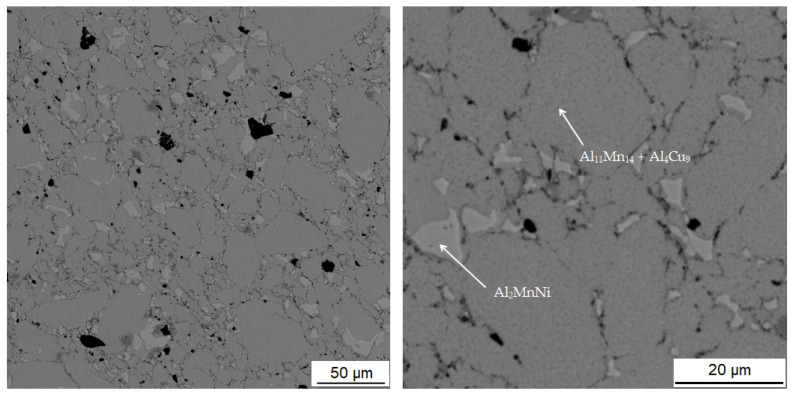
Microstructure of alloy with 100% excess of aluminum over stoichiometry sintered at 900 °C and heat-treated by annealing at 800 °C for 3 h.

Table 6 shows the composition of the two phases in sample sintered at 900 °C and then heat-treated by annealing at 800 °C for 3 h. First phase contains 39.2 at.% aluminum, 38.4 at.% manganese, 13.6 at.% silicon and 6.6 at.% iron. It can be seen from the microstructure in Figure 12 that the matrix again consists of two different phases. This is relatively well visible in Figure 6c. It is visible that the matrix consists of aluminum, manganese and silicon. Furthermore, a significant amount of copper is also present in this phase. From the results of X-ray diffraction analysis for alloy sintered at 900 °C and alloy sintered at 950 °C, which were processed in a similar way as sintered alloy, which was then heat-treated, it can be said that it is a two-phase matrix with a structure corresponding to Al_11_Mn_14_ and Al_4_Cu_9_.

The second phase is lighter in the BSE micrograph than the matrix and contains the most aluminum, which is 47.1 at.% in this phase. Furthermore, this phase contains 25.2 at.% manganese and 9.5 at.% nickel. Due to the fact that the phase has high contents of nickel and copper, it is the Al_2_MnNi phase.

### 3.4. Mechanical and Tribological Properties

Microhardness was measured in all states of processing. For the sample sintered at 900 °C and then heat-treated by annealing at 800 °C, this phase microhardness was not measured due to poor phase resolution.

In the sample in the reduced state, the matrix phase has a microhardness value of 748 ± 24 HV 0.1. Phase Al_2_MnNi in the same sample has a lower hardness of only 562 ± 28 HV 0.1. In the matrix of the sample sintered at 900 °C, the microhardness reached 903 ± 41 HV 0.1. Phase Al_2_MnNi in the same sample was lower at 785 ± 36 HV 0.1. The sample sintered at 900 °C and then heat-treated by annealing at 800 °C had the highest matrix microhardness, namely 1005 ± 33 HV 0.1. The reason is probably the dual-phase matrix. In the sample sintered at 950 °C, the matrix phase had a hardness of 979 ± 11 HV 0.1. Phase Al_2_MnNi reached a hardness of 760 ± 32 HV 0.1.

**Figure 13:**
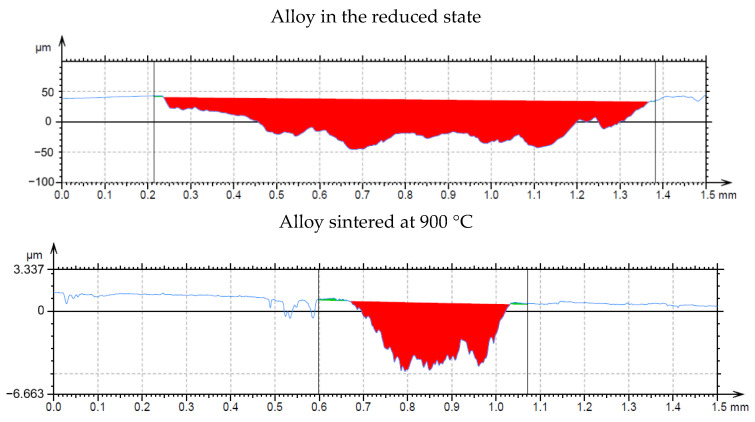
Wear track profile of alloy in the reduced state and the alloy sintered at 900 °C.

**Figure 14:**
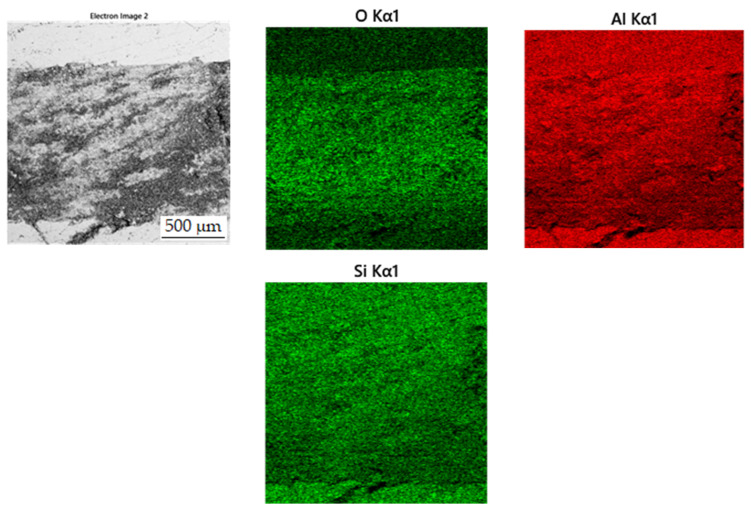
EDS map of the distribution of the elements of wear track in alloy in the reduced state.

**Figure 15:**
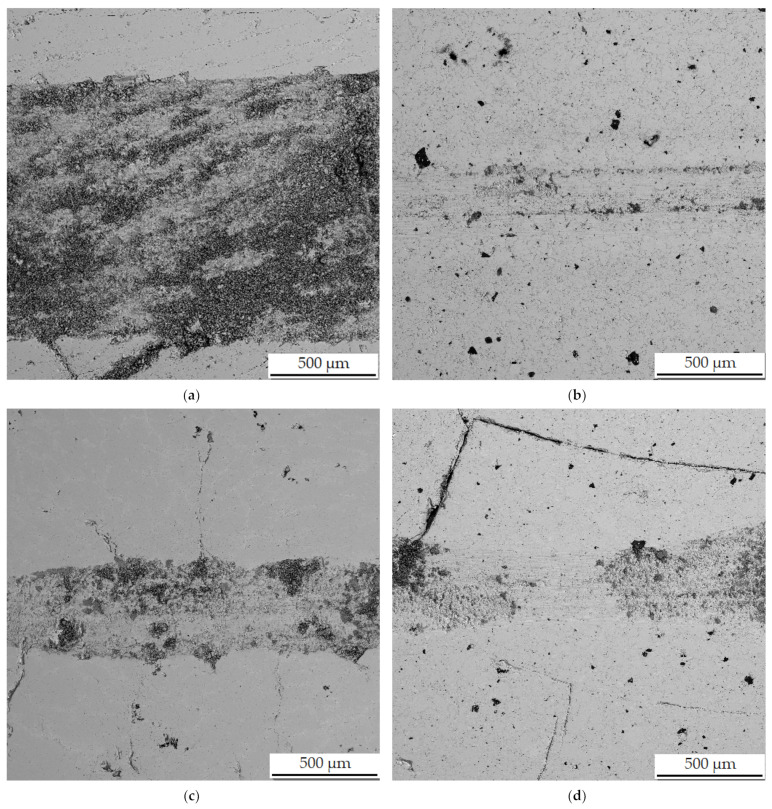
Wear tracks on aluminothermically prepared samples after testing with Al_2_O_3_ ball as the static partner. (**a**) Alloy with 100% excess of aluminum over stoichiometry in the reduced state; (**b**) alloy sintered at 900 °C; (**c**) alloy sintered at 950 °C; (**d**) alloy sintered at 900 °C and heat-treated by annealing at 800 °C for 3 h.

In the wear-rate test (Figure 13), there is a significant expansion of the wear track in sample in the reduced state. From the distribution of the elements in Figure 14, it can be seen that there is an apparent oxidation in the track, which can be seen mainly in the element map of oxygen. This is further visible in Figure 15a, where a darker color in the backscattered electron mode indicates the presence of elements with a lower oxidation number, in this case oxygen bound in oxides. The element map of nickel and copper shows that the Al_2_MnNi phase, rich in these two elements, occurs in this sample in a linear arrangement. The highest wear rate was reached by this sample, which had this rate about fifty times compared to sintered samples. The average coefficient of friction of the sample in the reduced state reached the highest value, namely 0.74, and the roughness of the sample was 1.330 µm (Table 7). All these reasons lead to faster wear of the sample in the as-reduced state and a greater susceptibility to particle breakage.

**Figure 16:**
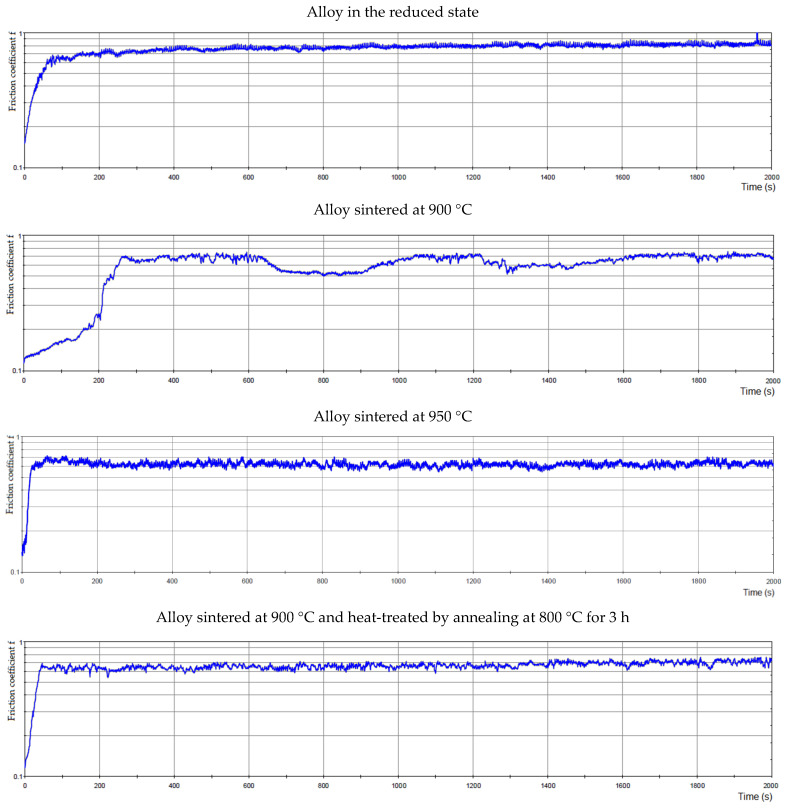
The friction coefficient of alloys depending on time.

The coefficient of friction depending on the time for all alloys is in Figure 16. However, the friction coefficients were identical for the alloy sintered at 900 °C and 950 °C, reaching the value of 0.62. For the alloy sintered at 950 °C, Figure 16 shows a smoother course than for the alloy sintered at 900 °C. The roughness of the alloy sintered at 900 °C was 0.078 µm, and for the alloy sintered at 950 °C the roughness was 0.017 µm. The sample sintered at 900 °C and then heat-treated by annealing at 800 °C showed a higher value of the average friction coefficient than sintered samples, namely 0.66. The roughness for this alloy was 0.151 µm. The values of the average friction coefficient of the sample are different depending on the type of sample preparation. This could be due to the fact that a higher hardness value was measured in alloy sintered at 900 °C and then heat-treated by annealing at 800 °C; thus, the matrix in this sample is harder and more brittle than in the other samples. As these more brittle particles are more easily broken out, there was a slight increase in the friction coefficient in this sample.

In all samples, which where sintered, the chains of the phase identified as Al_2_MnNi are broken by grinding, and that is why the predisposition to such a degree of oxidation and breakage of particles was not fulfilled as in alloy in the reduced state, which was not further crushed. Therefore, these samples do not have the same wear-track width (Figure 13) and oxidation does not occur to the same extent as in alloy in the reduced state. Sample sintered at 900 °C and sample sintered at 900 °C and then heat-treated have approximately the same wear-track width as sample sintered at 950 °C (Figure 13).

In alloy sintered at 950 °C, there is a slight increase in wear rate compared to samples sintered at 900 °C and samples sintered at 900 °C and then heat-treated. This is probably due to the fact both of those samples have a two-phase matrix with a structure corresponding to Al_11_Mn_14_ and Al_4_Cu_9_, while alloy sintered at 950 °C has a matrix Al_4_Cu_9_ with a smaller representation of the Al_11_Mn_14_ phase. A sample with a phase with Al_4_Cu_9_ structure, which proved to be softer above, is therefore more prone to abrasion.

## 4. Discussion

**Figure 17:**
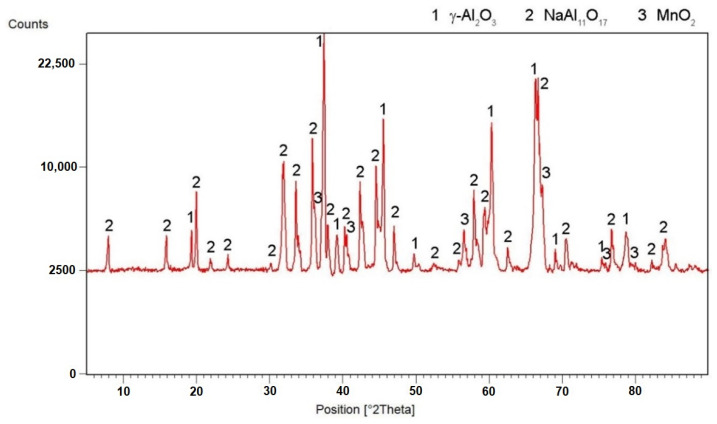
X-ray diffraction pattern of the slag.

A “natural” alloy from deep-sea nodules was prepared by aluminum reduction. This resulted in an alloy which, according to XRF analysis, contains about half the content of manganese and alloying elements such as aluminum, iron and others. Even though the idea was to prepare aluminum-based alloy by aluminothermic method directly, the result was still the manganese alloy. It could be stated that it is impossible to obtain aluminum alloy by this process, probably for the following reasons:

- The oxidation of the excessive aluminum occurred directly instead of melting. For this reason, it was transferred to the slag phase instead. This hypothesis was confirmed by the XRD analysis of the slag (Figure 17), where a high amount of the aluminum oxide was detected.
- The formed intermetallics contain high amounts of aluminum (up to approx. 47 at.%, see Table 3, Table 4, Table 5 and Table 6) and hence there is not enough aluminum to form a solid solution-based matrix.

In the reduced state, it was found by XRD analysis that the sample contained three main phases—tetragonal Al_11_Mn_14_ phase; cubic Al_2_MnNi high-aluminum ternary phase, which also dissolves nickel and copper; and minor Mn_2_P phase. The phase composition was completely different from our previous results on the characterization of the aluminothermically reduced alloys with the use of 0–20% excess amount of aluminum [22]. In these alloys, the matrix was changing from Mn_0.66_Ni_0.2_Si_0.14_ through β-Mn to α-Mn. In addition to these phases, the Mn_2_FeAl and Mn_2_FeSi Heusler phases were also determined in the case of the alloys prepared with the aluminum excess of 10–20%. The phase with the Al_4_Cu_9_ structure type, having the approximate stoichiometry of (Cu,Mn)_3_(Al,Si), was also detected in the alloys with 0–20 wt. % of aluminum [22]. All of the previously tested alloys were characterized in the state after reduction and remelting in vacuum-induction furnace. The conditions are probably relatively close to the reduced, crushed, sintered and annealed state in this work, because in both cases the material was molten already during the aluminothermic process. In addition, previous tests with the alloys reduced using small excesses of aluminum revealed no difference in phase composition between these states [22,27]. The phase-formation sequence probably continues with the increasing aluminum content as follows:

- manganese-based solid solution disappears;
- Mn_2_FeAl and Mn_2_FeSi phases are substituted by Al_2_MnNi phase, having very similar morphology;
- tetragonal Al_11_Mn_14_ phase forms, but it is converted partially to the cubic phase with Al_4_Cu_9_ structure during powder metallurgy processing.

The conditions for sintering were determined from the differential thermal analysis. From the obtained results of DTA analysis it can be stated that the endothermic peak shifts with a higher content of excess aluminum to higher onset temperatures. It is also possible to derive the processing temperature of the material in the case of the use of solid-phase sintering and so the material with a 100% excess of aluminum should be processed at temperatures up to approx. 950 °C. In comparison with previous results [22], the interval in which it is possible to work with this material is wider than in the case if lower-aluminum alloys. This is clear already from the binary Al-Mn phase diagram, where the increase of the solidus and liquidus temperature can be observed locally at the composition corresponding to our alloy [28]. However, the melting already occurred during sintering at 950 °C, probably due to local overheating or due to the localization of the temperature measurement, as discussed above. Sintering and the following heat treatment caused following changes in the phase composition:

- The tetragonal Al_11_Mn_14_ matrix phase continuously transformed to a phase with almost the same chemical composition, but with the cubic structure of the Al_4_Cu_9_ structure type. Because of the same chemical composition of both phases, it can be expected that this new phase is in fact just a different structure type of Al_11_Mn_14_ phase or a phase with very close chemical composition.
- The Mn_2_P phase disappeared and the phosphorus dissolved in the Al_11_Mn_14_ phase.

The most important effect of sintering was, however, the fragmentation of the intermetallics by crushing during milling. The following sintering resulted in a significantly reduced grain size. The hardness of individual samples increased slightly with higher sintering temperature. Hardness also increased with heat treatment. The matrix phase is always harder for the samples than the secondary phase, and its increase during sintering and heat treatment is connected with the increasing amount of the phase with the Al_4_Cu_9_ structure. The highest wear rate could be observed in the sample, which was only reduced with aluminum and was no longer crushed and ground. Thus, with crushing and grinding of the sample and subsequent sintering, the wear rate can be reduced due to a fragmentation of the brittle intermetallics. The sintering temperature as well as the heat treatment have negligible effect on the wear resistance. When measuring the wear rate, it was confirmed that the softer secondary phase of the sample breaks off more easily than the matrix phase of the sample. Because of the above-mentioned tribological properties, it can be stated that aluminothermically prepared alloys from the deep-sea nodules can be used as the protective coating for aluminum alloys. The connection between the layer and the alloy would be provided by the diffusion coefficient.

## 5. Conclusions

The aim of this work was the direct preparation of aluminum alloy by aluminothermic reduction of deep-sea nodules, possibly leading to the simple production of Al-Mn-based alloys. However, the as-reduced material composed of a series of aluminum-manganese-based intermetallics was obtained instead. The material is composed of the Al_11_Mn_14_ matrix phase, Al_2_MnNi phase, which is softer than the matrix, and a minor amount of Mn_2_P. During crushing, sintering and heat treatment, the Al_11_Mn_14_ matrix is partially transformed to a phase of the same composition and Al_4_Cu_9_ structure. The phosphide disappears during the above-mentioned processing, and phosphorus dissolves in Al_11_Mn_14_ phase. The most beneficial effect of powder metallurgy processing was the grain refinement due to crushing of the intermetallics. It also resulted in improvement of the wear rate of the alloy. Due to good tribological properties, the prepared alloy could be possibly used, e.g., as protective coatings for aluminum alloys in sliding contact with steel.

## Figures and Tables

**Table 1:** Chemical composition (XRF) of the deep-sea nodules (in wt. %) [25].

Element	Mn	Fe	Si	Al	Mg	Ca	Na	Cu	Ni	Ti	Zn	Co	O
[wt. %]	30.57	4.41	3.53	2.16	1.87	1.84	1.64	1.18	1.14	0.35	0.14	0.13	bal.

**Table 2:** Chemical composition of the alloy (in wt. %) prepared by reduction with a 100% excess of aluminum.

Element	Mn	Al	Fe	Si	Ni	Cu	Ti	Mo	P	Zr
[wt. %]	53.19	22.54	9.65	6.54	3.75	3.65	0.30	0.19	0.17	0.04

**Table 3:** Chemical composition of the individual phases in the sample in the reduced state.

	Mn	Al	Fe	Si	Cu	Ni	P	Ti	C	V
Al_11_Mn_14_ (at.%)	37.5	38.4	5.9	14.8	1.7	1.1	0.4	-	-	-
Al_2_MnNi (at.%)	32.1	43.5	5.6	7.8	5.1	5.9	-	-	-	-
Mn_2_P (at.%)	54.9	12.5	2.6	8.7	-	1.4	19.8	-	-	-
TiC (at.%)	3.7	5.8	-	-	-	0.5	-	35.7	49.6	4.7

**Table 4:** Chemical composition of individual phases in the sample sintered at 900 °C.

	Mn	Al	Fe	Si	Cu	Ni	P	C	Ti	V	O	Mg
Al_11_Mn_14_ + Al_4_Cu_9_ (at.%)	37.2	39.7	6.4	14.0	1.3	1.2	0.2	-	-	-	-	-
Al_2_MnNi (at.%)	31.2	44.0	7.6	8.2	3.2	5.7	-	-	-	-	-	-
TiC (at.%)	10.9	10.9	1.8	2.4	0.4	0.6	-	44.4	24.5	3.3	-	-
Al_2_O_3_ (at.%)	7.3	35.5	1.3	2.6	0.4	0.3	-	4.7	0.1	-	42.0	5.5

**Table 5:** Chemical composition of individual phases in sample sintered at 950 °C.

	Mn	Al	Fe	Si	Cu	Ni	P	Co
Al_4_Cu_9_ (at.%)	39.0	37.5	5.9	14.8	1.6	1.1	-	-
Al_2_MnNi (at.%)	31.3	42.7	7.2	7.4	4.5	6.4	-	0.5
Al_11_Mn_14_ (at.%)	39.4	37.0	5.8	14.7	1.5	1.2	0.4	-

**Table 6:** Chemical composition of individual phases in alloy sintered at 900 °C and heat-treated by annealing at 800 °C.

	Mn	Al	Fe	Si	Cu	Ni	Co
Al_11_Mn_14_ + Al_4_Cu_9_ (at.%)	38.4	39.2	6.6	13.6	1.2	1.0	-
Al_2_MnNi (at.%)	25.2	47.1	8.2	4.4	4.9	9.5	0.6

**Table 7:** Tribological properties of tested alloy against Al_2_O_3_ ball. Ra—roughness of the surface, f—friction coefficient, w—wear rate.

Alloy	Ra (µm)	f (Al_2_O_3_)	w (Al_2_O_3_) (mm^3^N^−1^m^−1^)
Alloy in the reduced state	1.330	0.74	$3.58\times 10^{-3}$
Alloy sintered at 900 °C	0.078	0.62	$6.30\times 10^{-5}$
Alloy sintered at 950 °C	0.017	0.62	$1.76\times 10^{-4}$
Alloy sintered at 900 °C and then heat-treated	0.151	0.66	$6.76\times 10^{-5}$

## Data Availability

Not applicable.
